# Mechanical stimulation of human dermal fibroblasts regulates pro-inflammatory cytokines: potential insight into soft tissue manual therapies

**DOI:** 10.1186/s13104-020-05249-1

**Published:** 2020-08-27

**Authors:** Aric Anloague, Aaron Mahoney, Oladipupo Ogunbekun, Taylor A. Hiland, William R. Thompson, Bryan Larsen, M. Terry Loghmani, Julia M. Hum, Jonathan W. Lowery

**Affiliations:** 1 Division of Biomedical Science, Marian University College of Osteopathic Medicine, 3200 Cold Spring Rd, Indianapolis, IN 46222 USA; 2 Bone and Mineral Research Group, Marian University, Indianapolis, IN 46222 USA; 3 Indiana Center for Musculoskeletal Health, School of Medicine, Indiana University, Indianapolis, IN 46202 USA; 4 Department of Physical Therapy, School of Health and Human Sciences, Indiana University, Indianapolis, IN 46202 USA

**Keywords:** Manual therapy, Osteopathic manipulative therapy, Massage, Soft tissue, Inflammation, Physiotherapy, Physical therapy, Myofascial release

## Abstract

**Objective:**

Soft tissue manual therapies are commonly utilized by osteopathic physicians, chiropractors, physical therapists and massage therapists. These techniques are predicated on subjecting tissues to biophysical mechanical stimulation but the cellular and molecular mechanism(s) mediating these effects are poorly understood. Previous studies established an in vitro model system for examining mechanical stimulation of dermal fibroblasts and established that cyclical strain, intended to mimic overuse injury, induces secretion of numerous pro-inflammatory cytokines. Moreover, mechanical strain intended to mimic soft tissue manual therapy reduces strain-induced secretion of pro-inflammatory cytokines. Here, we sought to partially confirm and extend these reports and provide independent corroboration of prior results.

**Results:**

Using cultures of primary human dermal fibroblasts, we confirm cyclical mechanical strain increases levels of IL-6 and adding long-duration stretch, intended to mimic therapeutic soft tissue stimulation, after cyclical strain results in lower IL-6 levels. We also extend the prior work, reporting that long-duration stretch results in lower levels of IL-8. Although there are important limitations to this experimental model, these findings provide supportive evidence that therapeutic soft tissue stimulation may reduce levels of pro-inflammatory cytokines. Future work is required to address these open questions and advance the mechanistic understanding of therapeutic soft tissue stimulation.

## Introduction

Soft tissue manual therapies such as massage and myofascial release are commonly utilized by osteopathic physicians, chiropractors, physical therapists and massage therapists [1–4]. These techniques are predicated on subjecting tissues to biophysical mechanical stimulation [5, 6]. While the precise cellular and molecular mechanism(s) mediating these effects are poorly understood, the limited available evidence suggests that soft tissue manual therapy may reduce inflammation [6]. For instance, a series of studies established an in vitro model system for examining therapy-informed mechanical stimulation of human dermal fibroblasts, which are a cell type that resides in close approximation to vasculature and lymphatics and are recipient of strain from soft tissue manual therapy (reviewed in [7]). This in vitro work demonstrated that strain intended to mimic repetitive, overuse injury of fibroblasts induces the secretion of numerous cytokines; reduces fibroblast proliferation rate; and increases fibroblast apoptosis. Moreover, mechanical strain intended to mimic soft tissue manual therapy reverses numerous aspects of this phenotype [8–11], including reduced secretion of pro-inflammatory interleukin (IL)-6, increased fibroblast proliferation, and reduced fibroblast apoptosis. These findings are generally consistent with prior work showing that soft tissue massage reduces levels of IL-6 in human soft tissue biopsies [12].

We sought to provide further insight and replicate a portion of the previous in vitro findings examining mechanical stimulation of dermal fibroblasts. Here, we provide independent corroboration that cyclical mechanical strain intended to mimic repetitive motion injury increases levels of IL-6 in conditioned media from dermal fibroblasts. Moreover, we confirm that adding long-duration stretch, intended to mimic therapeutic soft tissue stimulation, after cyclical strain reduces IL-6 levels and extend the prior work by reporting that it also reduces levels of the pro-inflammatory cytokine IL-8.

## Main text

### Materials and methods

#### Fibroblast culture and strain

**Fig. 1:**
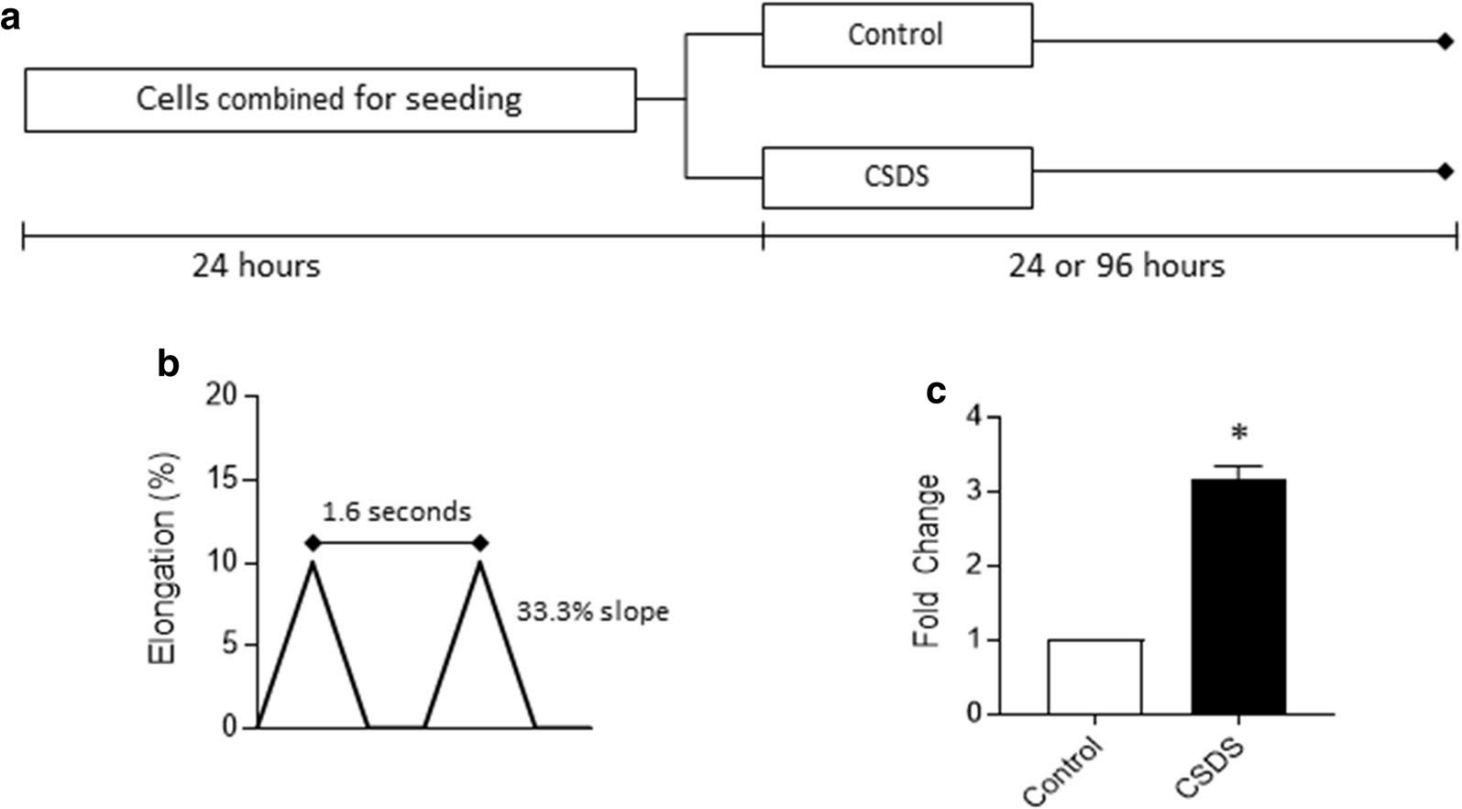
**a** Schematic representation of cell culturing method designed to allow matched comparison between unstrained primary human dermal fibroblasts (Control) and primary human dermal fibroblasts subjected to cyclic short-duration strain (CSDS). All conditioned media was collected simultaneously 24 or 96 h after the onset of the CSDS strain profile. **b** Schematic representation of CSDS profile used for studies in Fig. 1 and 2. **c** ELISA for interleukin (IL)-6 collected from primary dermal fibroblasts 96 h following CSDS compared to time-matched unstrained controls. Data are mean ± SEM normalized to Control; n = 3 per condition. * indicates p < 0.05 against Control by paired t-test

**Fig. 2:**
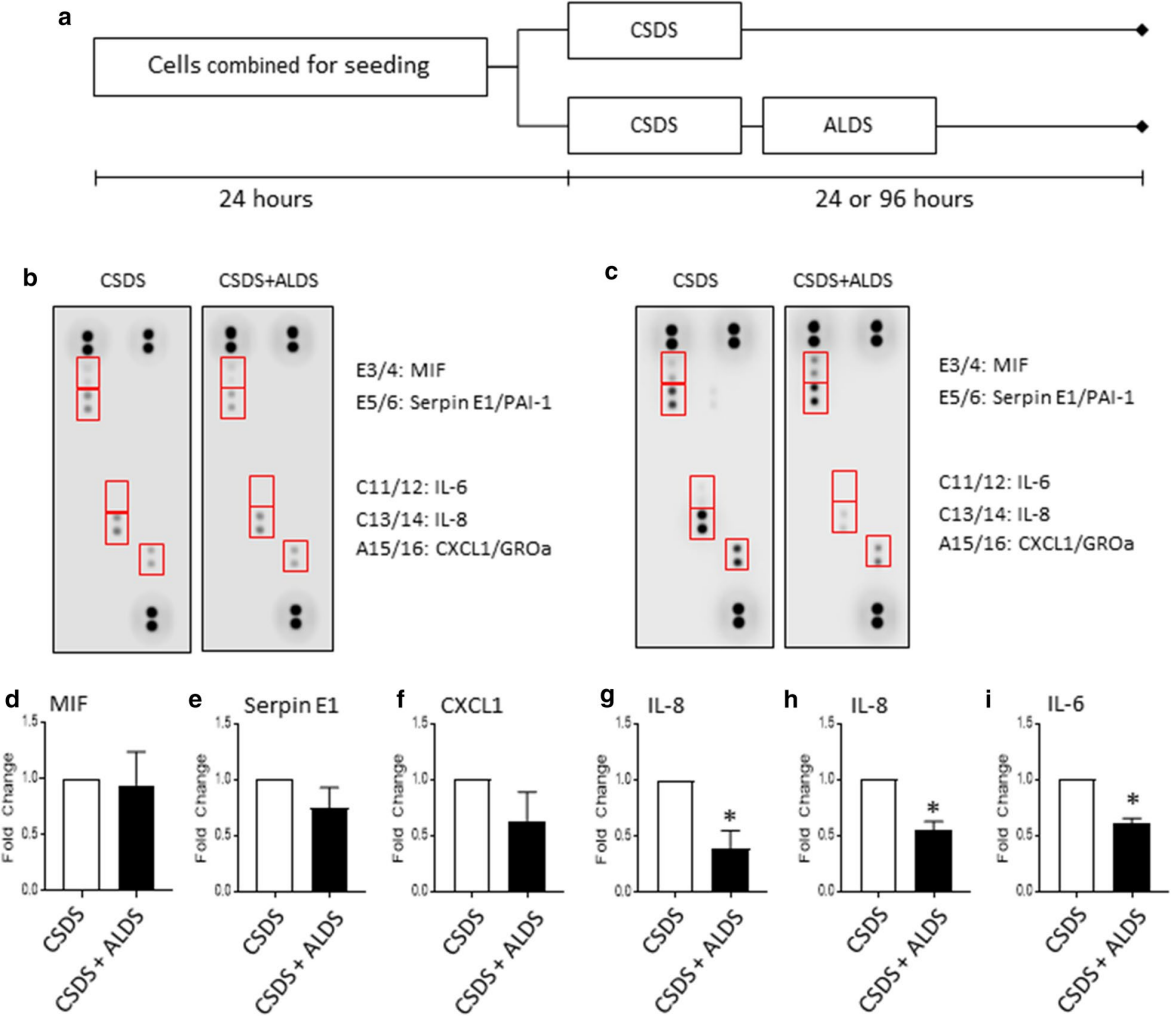
**a** Schematic representation of cell culturing method designed to allow matched comparison between primary human dermal fibroblasts subjected to cyclic short-duration strain (CSDS) or CSDS combined with acyclic long-duration strain (ALDS). All conditioned media was collected simultaneously 24 or 96 h after the onset of the CSDS strain profile. **b**, **c** Multi-analyte cytokine membrane array analyses on conditioned media collected 24 h (**b**) or 96 h (**c**) after the onset of CSDS strain profile. Red boxes indicate the membrane region corresponding to macrophage migration inhibitory factor (MIF), Serpin E1/Plasminogen activator inhibitor (PAI-1), interleukin (IL)-6, IL-8, and chemokine (C-X-C motif) ligand 1 (CXCL1)/Growth-regulated oncogene (GRO)-α. **d–g** Quantification of cytokine membrane array analyses for MIF (D), Serpin E1 (E), CXCL1 (F), and IL-8 (G). Data are mean ± SEM normalized to CSDS; n = 4 per condition. * indicates p < 0.05 against CSDS by paired t-test. **h**, **i** Quantification of cytometric bead array analyses for IL-8 (H) and IL-6 (I). Data are mean ± SEM normalized to CSDS; n = 3 per condition. * indicates p < 0.05 against CSDS by paired t-test

**Fig. 3:**
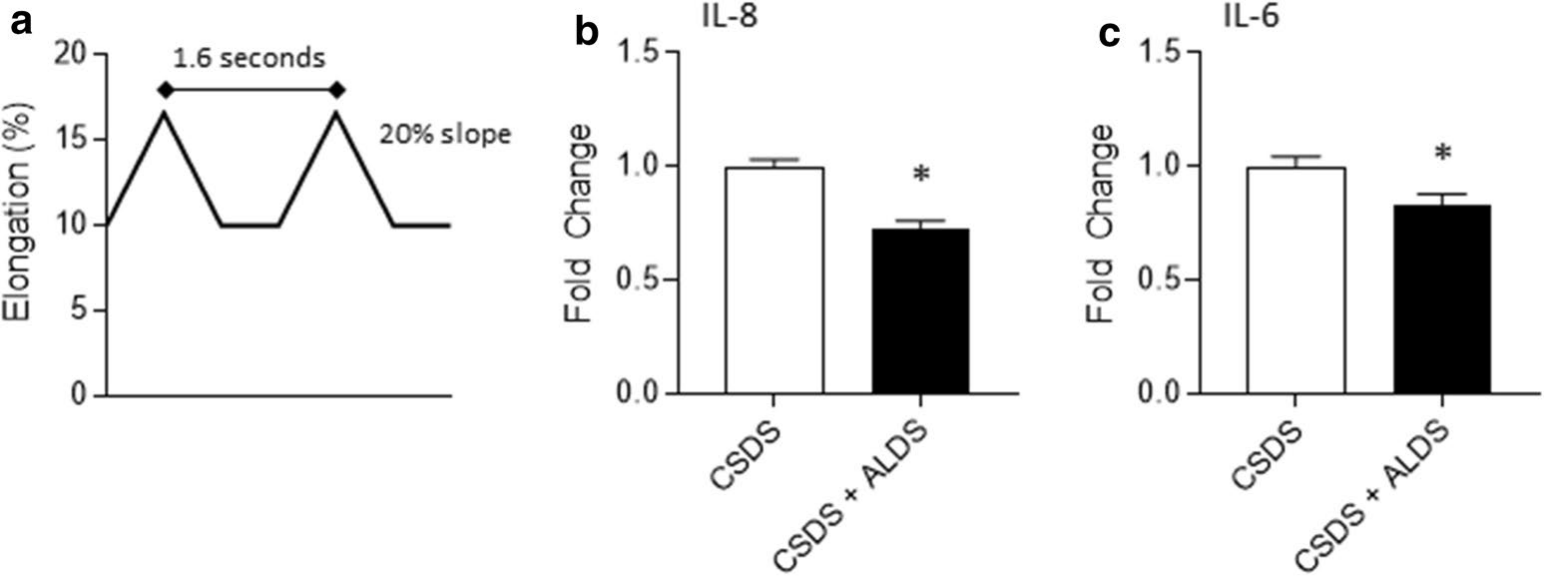
**a** Schematic representation of the second cyclic short-duration strain (CSDS) profile utilized for studies in Fig. 3. **b**, **c** Determination of IL-8 (**b**) and IL-6 (**c**) levels in conditioned media collected from primary human dermal fibroblasts 96 h following onset of CSDS profile. Data are mean ± SEM normalized to CSDS; n ≥ 8 per condition. * indicates p < 0.05 against CSDS by unpaired t-test

Primary human dermal fibroblasts (#PCS-201-012) were purchased from American Type Culture Collection (ATCC, Manassas, Virginia, USA) and cultured as directed by the vendor. Cells were free of mycoplasma contamination as confirmed by the MycoProbe Mycoplasma Detection Kit (R&D Systems, Minneapolis, Minnesota, USA) used as directed by the manufacturer. Using a parallel study design as detailed in Fig. 1a and Fig. 2a, cells were combined from separate flasks then seeded at 120,000 cells per well on 6-well flexible collagen I-coated membranes (Flexcell International, Burlington, North Carolina, USA). The next day, cells were left unstrained (control) or mechanical stimulation was performed on a Flexcell FX-6000 according to two previously reported strain profiles [6]. Briefly, for the first cyclic short-duration strain (CSDS) profile, fibroblasts were subjected to an 8-h cycle with 1.6 s bouts of deformation increasing at 33.3%/second starting from rest to a maximum of 10% beyond resting length, followed by decreasing strain to baseline at 33.3%/second (Fig. 1a). For the second CSDS profile, fibroblasts were subjected to an 8-h cycle with 1.6 s bouts of deformation increasing at 22%/second starting at a baseline strain of 10% and a maximum of 16.6%, followed by decreasing strain to baseline at 22%/second (Fig. 3a). For acyclic long-duration strain (ALDS), after a 3-h rest period following CSDS, cells were subjected to a single 60 s bout of stretch at 6% beyond resting length at a loading rate of 3%/second followed by release at 1.5%/second until return to resting length. Conditioned media was collected simultaneously from all samples either 24 h or 96 h after initiation of the CSDS strain protocol and stored at − 80 °C.

### IL-6 and IL-8 enzyme-linked immunoassays

The collected conditioned media was analyzed using ELISA kits for human IL-6 and IL-8 (ThermoFisher Scientific, Waltham, Massachusetts, USA) to determine the concentration of these respective proteins. The ELISA methods were performed following instructions provided by the manufacturer and quantified on a FluoStar OPTIMA (BMG, Cary, North Carolina, USA). Since concentrations for IL-6 and IL-8 varied between runs, data were normalized to either unstrained control (Fig. 1c) or CSDS strain profile (Fig. 2h–i) for each paired flex run.

### Human cytokine membrane array

Conditioned media was analyzed using the Proteome Profiler Human Cytokine Array (R&D Systems) as directed by the manufacturer. The arrays were developed using WesternBright Sirius reagent (Advansta, San Jose, California, USA) on a C-Digit scanner (LI-COR, Lincoln, Nebraska, USA) and signal densities were determined using Image Studio software package (LI-COR). Data were normalized to CSDS strain profile for each paired flex run.

### Cytometric bead array

Conditioned media was analyzed with the Human Pro-Inflammatory Cytokine Cytometric Bead Array kit (BD Biosciences, Franklin Lakes, New Jersey, USA) as directed by the manufacturer using a Accuri C6 Flow Cytometer (Becton, Dickinson and Company, Franklin Lakes, New Jersey, USA). Since concentrations for IL-6 and IL-8 varied between runs, data were normalized to CSDS strain profile for each paired flex run.

### Statistical analyses

Statistical analyses were performed using GraphPad Prism 5 as described in each respective figure legend or in the text. A p-value of < 0.05 was considered significant.

## Results

We employed a parallel study design to examine the effects of different mechanical strain profiles on cytokine levels in conditioned media from primary human dermal fibroblasts (Fig. 1a and 2a); cells for each condition were seeded on collagen I-coated flexible membranes in separate 6-well dishes from a single stock and, regardless of experimental condition, were maintained in the incubator simultaneously. Cells were placed on the FlexCell device (which resides in the same incubator) for mechanical stimulation then returned to the shelf. At the conclusion of the experiment, conditioned media was collected from each plate simultaneously to allow matched observations of cytokine levels between experimental conditions.

First, to replicate conditions of prior reports [8, 13, 14], fibroblasts were subjected to a mechanical force profile intended to mimic repetitive motion injury (i.e., cyclic short-duration strain, CSDS) used in multiple studies [11, 13, 14] wherein cells were repeatedly stretched between baseline and 10% beyond resting length every 1.6 s for 8 h (Fig. 1b). We first collected conditioned media from the control and strained cells at 24 h and performed ELISA for IL-6 levels; this limited, single observation pilot run (n = 1 plate per condition) was consistent a prior report [8] in showing that CSDS results in approximately 2.5× higher levels of IL-6 compared to unstrained cells (control: 3.34 pg/ml; CSDS: 9.41 pg/ml). However, since this finding at 24 h post-CSDS is not consistent across all reports (see [13, 15]), we extended the culture period following CSDS to 96 h. This revealed threefold higher IL-6 levels in conditioned media obtained from fibroblasts subjected to CSDS compared to unstrained controls (Fig. 1c), which is consistent with a prior report [13].

Having successfully established the model system in our lab, we next subjected primary human dermal fibroblasts to CSDS or CSDS followed by mechanical strain intended to mimic therapeutic soft tissue stimulation (i.e., acyclic long-duration strain, ALDS), such as massage or myofascial release (Fig. 2a). We then took a limited, single observation pilot run (n = 1 plate per condition) at 24 h post-initiation of CSDS strain and performed a multi-analyte cytokine membrane array that evaluates levels of thirty-six different cytokines simultaneously. This assay detected macrophage migration inhibitory factor (MIF), Serpin E1/Plasminogen activator inhibitor (PAI)-1, IL-8, and chemokine (C–X–C motif) ligand 1 (CXCL1)/Growth-regulated oncogene (GRO)-α in conditioned media from fibroblasts under both experimental conditions but the levels did not differ between conditions at this time point (Fig. 2b). Notably, IL-6 was not detected in this assay (Fig. 2b) but was detectable by ELISA; consistent with the other cytokines, IL-6 levels did not differ in conditioned media from CSDS and CSDS + ALDS samples at this time point (data not shown). The same targets were detectable on cytokine membrane arrays from samples obtained 96-h following the initiation of CSDS (Fig. 2c). Consistent with the 24-h pilot run, there were no differences in levels of MIF, Serpin E1 or CXCL1 between CSDS and CSDS + ALDS samples (Fig. 2d–f). In contrast, IL-8 levels were significantly lower in CSDS + ALDS samples compared to CSDS alone samples by membrane array (Fig. 2g) and by secondary analysis via high-sensitivity cytometric bead array (Fig. 2h). Similarly, IL-6 levels, though undetectable by membrane array (Fig. 2c), were lower in CSDS + ALDS samples compared to CSDS alone samples by cytometric bead array (Fig. 2i), which is consistent with prior reports [7].

Next, in a separate set of studies, we examined a different CSDS profile that has also been used in the literature [8] wherein primary human dermal fibroblasts were cyclically stretched 10% beyond resting length to 16% beyond resting length every 1.6 s for 8 h (Fig. 3a). We focused our analyses on IL-8 and IL-6 by cytometric bead arrays, which revealed lower levels of both cytokines in CSDS + ALDS samples compared to CSDS alone samples (Fig. 3b, c).

## Conclusions

This study was designed to replicate and extend prior work using an in vitro model to examine the impact of mechanical stimulation of dermal fibroblasts, which are a cell type that is recipient of mechanical forces during therapeutic soft tissue manipulation (reviewed in [7]). Our findings corroborate the observation that ALDS, intended to mimic soft tissue therapy such massage, following CSDS reduces levels of the pro-inflammatory cytokine IL-6. Additionally, we provide the first evidence that ALDS following CSDS also reduces levels of the pro-inflammatory cytokine IL-8. We were unable to replicate the prior result that ALDS reduces expression of pro-inflammatory IL-1β as this cytokine was not detected in any of our assays.

## Limitations

Collectively, these findings provide supportive evidence that therapeutic soft tissue massage may reduce inflammatory cytokines and may assist in designing future mechanistic studies in this area. However, there are several important limitations to the in vitro model used by us and others that may temper the generalization of these results. For instance, our uniculture model examines only one soft tissue cell type as opposed to the complex tissue-level interactions that likely occur in vivo. Current work in our laboratory is examining the effects of mechanical stimulation on other clinically-relevant soft tissue cell types including skeletal muscle myocytes and satellite cells, adipocytes, and vascular endothelial cells. Similarly, we are unable to speculate how our findings might compare to a three-dimensional cell culture model as opposed to a cell in a monolayer. It is also unclear why reduced IL-6 and IL-8 levels were not observed at earlier time points in our pilot experiments or, importantly, if this delayed effect is clinically relevant or an artifact of the in vitro setting. That said, our results are highly consistent with prior work and provide independent confirmation that mechanical stimulation, as delivered here, alters pro-inflammatory cytokine release from dermal fibroblasts, which is consistent with the finding that IL-6 levels are lower in heterogenous soft tissue biopsies obtained from humans 2.5 h post-massage [12].

Finally, it is important to point out that, with regard to clinical relevance, soft tissue manual therapy likely involves several factors beyond mechanical stimulation, including sensory, cognitive, thermal, neurovascular, lymphatic, autonomic, neuro-hormonal-endocrine, psychosocial, and emotional components. Ultimately, the complex integration of multiple elements determines the biological response, functional outcomes, subjective pain perception, and sense of well-being associated with soft tissue manual therapies in clinical care. Future work is required to address these open questions and advance the mechanistic understanding of therapeutic mechanical stimulation of soft tissues.

## Data Availability

The datasets used and/or analysed during the current study are available from the corresponding author on reasonable request.

## Abbreviations

CSDS: Cyclic short-duration strain
ALDS: Acyclic long-duration strain
IL: Interleukin
TNF: Tumor necrosis factor
